# A86 CANNABIS USE IN PATIENTS WITH INFLAMMATORY BOWEL DISEASE IS HIGHER FOLLOWING LEGALIZATION OF CANNABIS IN CANADA AND IS ASSOCIATED WITH LOWER QUALITY OF LIFE

**DOI:** 10.1093/jcag/gwac036.086

**Published:** 2023-03-07

**Authors:** V Iablokov, N Chande, T Ponich, V Jairath, J Gregor, R Khanna, S Asfaha

**Affiliations:** Division of Gastroenterology, Department of Medicine, Western University, London, Canada

## Abstract

**Background:**

Patients with inflammatory bowel disease (IBD), including Crohn’s disease (CD) and ulcerative colitis (UC), often experience fluctuating and unpredictable symptoms. Most individuals require chronic therapy with immunomodulators or novel biologics to maintain disease remission. In addition to conventional medical therapy, many patients also seek out alternative therapies such as cannabis. Reports in the USA suggest that cannabis is used by ~12% of UC and ~16% of CD patients, despite it being legally prohibited.

**Purpose:**

The aim of our study is to evaluate the use of cannabis in a cohort of patients with IBD following its legalization in Canada, and to assess its effects on IBD disease severity.

**Method:**

We conducted a prospective cohort study of adult IBD patients seen in clinic at a tertiary care center in London, ON. Patients completed an online 40-question survey that collected data on demographics, IBD disease history, cannabis use, and included the Short Inflammatory Bowel Disease Questionnaire (SIBDQ). The survey was distributed and collected by the REDCap platform maintained by Western University. The study was approved by the Western University Ethics Committee.

**Result(s):**

Completed surveys were obtained from 254 individuals (148 individuals with CD, 90 with UC and 16 with indeterminate colitis). Over half of participants were between 35-64 years of age and female. Fifty-three percent of participants reported life-time cannabis use and 51% of users started using cannabis only in the preceding 3 years. Individuals with CD had higher rates of recent use, defined as use within the past 6 months, when compared with UC (41% vs 31%). Cannabis was taken multiple times per week by 57% of users. Cannabis was used to treat GI symptoms by 30% of users, as well as to help with sleep (26%) and for recreation (27%). Despite side effects such as dry mouth, anxiety and concentration issues, 79% of users felt the benefits of cannabis outweighed its harms. Interestingly, only 46% of cannabis users discussed their use with their family physician or gastroenterologist. Recent cannabis users did not differ in the use of IBD medication or self-reported rates of GI symptoms. Furthermore, recent users did not differ in the rates of surgical procedures for IBD (recent 35% vs non-recent 32%). Recent cannabis users did have a significantly lower quality of life as indicated by SIBDQ scores when compared to non-recent users (recent use 37 vs non-recent use 40).

**Conclusion(s):**

Cannabis use among patients with IBD after its legalization is more than double the rate previously reported in the literature. Importantly, physicians are likely to be unaware of its prevalence in their practice. Cannabis was used by patients to treat GI and non-GI symptoms, and it was associated with lower SIBDQ scores. Our results suggest that physicians should inquire about Cannabis use amongst their patients with IBD, and that further studies are required to determine its effects on disease severity.

**Please acknowledge all funding agencies by checking the applicable boxes below:**

CIHR

**Disclosure of Interest:**

None Declared

